# Use of new recombinant proteins for ovarian stimulation in ruminants

**DOI:** 10.1590/1984-3143-AR2023-0092

**Published:** 2023-09-04

**Authors:** Pietro Sampaio Baruselli, Laís Ângelo de Abreu, Bruna Lima Chechin Catussi, Ana Carolina dos Santos Oliveira, Lígia Mattos Rebeis, Emanuele Almeida Gricio, Sofía Albertini, José Nélio Sousa Sales, Carlos Alberto Rodrigues

**Affiliations:** 1 Departamento de Reprodução Animal, Faculdade de Medicina Veterinária e Zootecnia, Universidade de São Paulo, São Paulo, SP, Brasil; 2 Universidade Federal de Juiz de Fora, Juiz de Fora, MG, Brasil; 3 Clínica Veterinária SAMVET, São Carlos, SP, Brasil

**Keywords:** follicle stimulating hormone, luteinizing hormone, bovine somatotropin

## Abstract

Currently, gonadotropin products (follicle stimulating hormone, FSH, and luteinizing hormone, LH) used in animal reproduction are produced by extraction and purification from abattoir-derived pituitary glands. This method, relying on animal-derived materials, carries the potential risk of hormone contamination and pathogen transmission. Additionally, chorionic gonadotropins are extracted from the blood of pregnant mares (equine chorionic gonadotropin; eCG) or the urine of pregnant women (human chorionic gonadotropin; hCG). However, recent advancements have introduced recombinant gonadotropins for assisted animal reproduction therapies. The traditional use of FSH for superovulation has limitations, including labor requirements and variability in superovulation response, affecting the success of *in vivo* (SOV) and *in vitro* (OPU/IVEP) embryo production. FSH treatment for superstimulation before OPU can promote the growth of a homogenous follicular population and the recovery of competent oocytes suitable for IVEP procedures. At present, a single injection of a preparation of long-acting bovine recombinant FSH (rFSH) produced similar superovulation responses resulting in the production of good-quality *in vivo* and *in vitro* embryos. Furthermore, the treatment with eCG at FTAI protocol has demonstrated its efficacy in promoting follicular growth, ovulation, and P/AI, mainly in heifers and anestrous cows. Currently, treatment with recombinant glycoproteins with eCG-like activity (r-eCG) have shown promising results in increasing follicular growth, ovulation, and P/AI in cows submitted to P4/E2 -based protocols. Bovine somatotropin (bST) is a naturally occurring hormone found in cows. Recombinant bovine somatotropin (rbST), produced through genetic engineering techniques, has shown potential in enhancing reproductive outcomes in ruminants. Treatment with rbST has been found to improve P/IA, increase donor embryo production, and enhance P/ET in recipients. The use of recombinant hormones allows to produce non-animal-derived products, offering several advantages in assisted reproductive technologies for ruminants. This advancement opens up new possibilities for improving reproductive efficiency and success rates in the field of animal reproduction.

## Introduction

Assisted reproductive technologies (ARTs) have transformed the dairy and beef production industries worldwide by revolutionizing their approach to breeding, reproductive efficiency and genetic selection, leading to significant improvements in the quality and quantity of animal products (Wiltbank et al., 2002; Bó et al., 2002; Baruselli et al., 2004c). The most used ARTs in the cattle industry, such as artificial insemination (AI), *in vitro* embryo production (IVEP), and embryo transfer (ET), have enabled more efficient breeding programs, allowing farmers and breeders to produce offspring with desirable traits such as improved milk production, meat quality, and disease resistance (Bó et al., 2007; Rodriguez-Martinez, 2011).

Ovarian pharmacological manipulation plays a crucial role in ARTs. In the cattle industry, hormonal treatments have a significant role in the control and manipulation of reproductive processes, ultimately leading to improved fertility. FSH, LH, hCG and eCG are commonly used to induce the development of follicles in the ovaries and ovulation, and to improve fertility in cows (Bó et al., 2006; Baruselli et al., 2006; Baruselli et al., 2008b; Marques et al., 2012). Bovine somatotropin (bST) is a hormone used to promote growth, enhance milk production in dairy cows, and optimize feed efficiency and reproductive outcomes (Cushman et al., 2001; Thatcher et al., 2006). Nevertheless, these hormones are produced by extraction and purification from abattoir-derived pituitary glands or extracted from fluids (blood and urine) of pregnant females. Nowadays, recombinant hormones, which are synthetic versions of hormones normally produced by animals (McClamrock, 2003), are produced using genetic engineering techniques. These involve inserting the genes responsible for producing the hormone into a host organism, such as a virus, bacteria, or yeast, allowing for the large-scale production of hormones for various applications (Sanderson and Martinez, 2020). Therefore, the utilization of recombinant hormones has revolutionized the large-scale production of non-animal-derived products, characterized by a remarkable purity and consistent composition. This progress enables diverse applications across industries without variability (Lunenfeld et al., 2019).

The combined use of ARTs and hormonal treatments has resulted in significant advancements in the cattle industry, enabling the production of a large number of animals with desirable traits, ultimately benefiting both farmers and consumers (Baruselli et al., 2018). The goal of the present review is to provide the reader with an update on the use of recombinant hormones for ovarian stimulation in ruminants with special emphasis on ARTs programs.

## Recombinant treatment (FSH-Like) for superovulation

Follicle stimulating hormone (FSH) is a crucial glycoprotein that exists as a heterodimer composed of two subunits, α and β. FSH plays a fundamental role in regulating gonadal functions, specifically stimulating the growth of follicles in ovaries and primary spermatocytes in the testes (Fortune et al., 2001). Initially, FSH formulations were extracted from the pituitary glands of pigs (pFSH) and sheep (oFSH) and contained both FSH and LH (Greep et al., 1942; Steelman et al., 1956). However, subsequent studies revealed that high concentrations of LH could negatively impact bovine embryo quality and production (Donaldson et al., 1986; Gonzalez et al., 1990; Mapletoft and Pierson, 1993).

The success of *in vivo* (SOV) and *in vitro* (OPU/IVEP) embryo production has been hindered by labor-intensive FSH treatments and variability in superovulation responses. Traditional superstimulatory treatments involve twice-daily intramuscular (IM) injections of pFSH for both *in vivo* (Bó et al., 2002) and *in vitro* (Vieira et al., 2014) embryo production. The need for frequent applications is due to the short half-life of FSH. From plasma profiles, the half-life and the disappearance of pFSH were estimated at 5 h and at 10 to 12 h, respectively (Demoustier et al., 1988). This necessitates precise handling and attention during the treatments to induce ovarian superstimulation effectively. Thus, implementing traditional superstimulatory protocols in large-scale programs can lead to failures and poor results due to the complexity and potential for errors during handling. Therefore, there is a demand for simplified protocols that can be efficiently and easily applied in the field, reducing handling and the incidence of potential errors. To address this, studies have focused on developing alternative methods to maintain FSH release for a prolonged period, aiming to improve the efficiency and success of superovulation protocols.

An alternative that has been studied for *in vivo* (Bó and Mapletoft, 2020) and *in vitro* (Vieira et al., 2016) embryo production is the use of a single injection of pFSH in a hyaluronan (HA) solution. HA, also known as hyaluronic acid, is a simple glycosaminoglycan with remarkable biocompatibility, and when used as a diluent, it facilitates a sustained release of various drugs. Overall, a single IM treatment of pFSH in a 2.0% solution of HA resulted in a similar number of transferable embryos as the traditional twice-daily IM protocol (Vieira et al., 2016; Bó and Mapletoft, 2020). The use of HA as a carrier for pFSH provides an efficient and effective means of achieving superovulation, making it a viable option for improving the success of both *in vivo* and *in vitro* embryo production procedures.

In parallel, recombinant DNA technology has emerged as a significant pharmacological advancement for producing FSH (rFSH). This technique utilizes biological processes capable of encoding the two subunits of FSH, resulting in the creation of recombinant forms of the hormone (Le Cotonnec et al., 1994; Calder et al., 2003). The rFSH can induce a superovulatory response without exogenous LH (Wilson et al., 1993). However, one of the main challenges in producing these recombinant proteins has been achieving proper post-translational modifications, particularly glycosylations (Hesser et al., 2011), which play a crucial role in extending the biological activity and half-life of the hormone. Proper glycosylation helps preserve the hormone from enzymatic degradation in the bloodstream, reducing renal and hepatic clearance (Morell et al., 1971; Sinclair and Elliott, 2005).

Traditionally, superstimulation of the ovaries using pFSH required multiple administrations at 12-hour intervals for four days (Monniaux et al., 1983). Nowadays, treatment with rFSH for SOV protocol improved the number of corpora lutea and embryo-derived compared to pFSH (Gutiérrez-Reinoso et al., 2023). Furthermore, recent studies have shown promising results with long-acting bovine recombinant FSH (brFSH). This long-acting version of rFSH has the potential to reduce the number of treatments and improve the efficiency of superovulation (SOV) protocols for *in vivo* (Carvalho et al., 2014) and *in vitro* embryo production (OPU/IVEP) (Rodrigues et al., 2023a, b). The use of a single-dose of long-acting brFSH simplifies donor management and enhances the efficiency of embryo transfer programs. Additionally, studies comparing Holstein heifers treated with multiple doses of conventional pFSH administered twice daily during the SOV protocol with Holstein heifers receiving a single treatment of long-acting brFSH showed similar superovulation responses, resulting in the production of good-quality embryos in both groups (Carvalho et al., 2014).

FSH treatment for superstimulation before OPU has proven to be beneficial in promoting the growth of a homogenous population of follicles and facilitating the recovery of competent oocytes suitable for *in vitro* embryo production (IVEP) procedures. Studies conducted in cattle (Blondin et al., 2002; Vieira et al., 2014) and buffalo (Carvalho et al., 2019) have shown that FSH treatment leads to the development of follicles with larger diameters, which, in turn, improves the quality of the oocytes obtained and enhances the efficiency of the IVEP procedures. The growth of follicles to larger diameters has been associated with the acquisition of developmental potential in oocytes, enabling them to reach the blastocyst stage during embryo development (Seneda et al., 2001). It has been observed that the developmental competence of the oocyte continues to increase as the follicular diameter approaches the LH surge (Sirard, 2012). The process of follicular growth and oocyte development is governed by a sequence of molecular and transcriptional alterations. The quality and developmental potential of the oocyte have been linked to these molecular changes during follicular growth (Labrecque and Sirard, 2014). Consequently, oocyte quality is highly correlated with the diameter of the follicle in which it matures.

In Brazil, studies have been conducted to assess the impact of follicular stimulation using long-acting brFSH on the efficiency of OPU and IVEP in Holstein heifers. The effects of treatment on follicular diameter, viable oocyte rate, recovery rate, cleavage rate, and blastocyst rate were evaluated (Rodrigues et al., 2023a). Heifers in the Control group (n=30) received no further treatment, whereas heifers in the brFSH group received a single dose of 50 µg (n=30) or 100 µg (n=30) of brFSH 3 days before OPU. There was no difference between groups in the total number of follicles aspirated per OPU session (Control=13.2±1.4 vs. 50 µg=11.9±1.2 vs. 100 µg=11.6±0.9; P=0.90), however, treatment with brFSH regardless of dosage (50 µg or 100 µg) increased the number of medium follicles (6-10 mm) and decreased the number of small follicles (<6 mm) compared to the Control group at OPU. Furthermore, heifers treated with 100 µg of brFSH produced a greater number of large follicles (>10 mm) than the Control group. Heifers treated with 100 µg of brFSH had greater (P=0.004) viable oocytes rate than the Control group (Control=54.0%^b^ vs. 50 µg=62.0%^ab^ vs. 100 µg=71.0%^a^; P=0.004), without compromising the recovery rate (Control=79.7% vs. 50 µg=72.7% vs. 100 µg=75.5%; P=0.57). Heifers treated with 100 µg of brFSH also exhibited higher cleavage (Control=30.9%^b^ vs. 50 µg=41.4%^ab^ vs. 100 µg=52.3%^a^; P=0.02) and blastocysts rates (Control=7.1%^b^ vs. 50 µg=16.2%^ab^ vs. 100 µg=17.4%^a^; P=0.05), indicating improved embryo development potential compared to the Control group. The number of embryos produced per OPU was similar between groups (Control=0.97±0.22 vs. 50 µg=1.67±0.40 vs. 100 µg=1.37±0.28; P=0.29). However, treatment with a single dose of brFSH did positively affect several parameters, such as follicular diameter and, viable oocyte, cleavage and blastocyst rates, without compromising the recovery rate, leading to an enhancement in the efficiency of OPU and IVEP procedures in Holstein heifers.

In another study conducted by Rodrigues et al. (2023b), lactating Holstein cows were subjected to different treatments to evaluate their impact on OPU and IVEP. One group received a single dose of 100 µg of brFSH 3 days before OPU (n=31), another group received a single dose of 150 µg of brFSH 3 days before OPU (n=33), and a third group received a total dosage of 200 mg of pFSH (Folltropin, Vetoquinol) administered in four decreasing doses over a 12-hour interval (57, 57, 43, and 43 mg; n=27). The total number of follicles aspirated did not differ between the groups (mean of 12.6±0.98; P=0.21). However, both brFSH treatments (100 µg and 150 µg) and the pFSH treatment resulted in an increase in the number of large follicles (>10 mm) and a decrease in the number of small follicles (<6 mm) at the time of OPU compared to the Control group (no FSH treatment; P<0.0001). Donors treated with 100 µg (71.1%) and 150 µg (74.7%) of brFSH exhibited similar recovery rates [number of cumulus-oocyte complexes (COCs) recovered/total number of follicles aspirated] compared to the control group (81.9%). However, donors treated with pFSH (68.0%) showed a lower oocyte recovery rate (P<0.05) than the control group. Previous studies have suggested a negative correlation between follicle size and recovery rate in donor cows and heifers, despite the improvement in oocyte quality with follicle growth (Seneda et al., 2001; Vieira et al., 2014). Furthermore, Rodrigues et al. (2023b) found that the blastocyst rate (number of blastocysts/number of oocytes cultured) was higher in donors treated with 150 µg of brFSH (27.4%) compared to those treated with 100 µg of brFSH (14.1%; P=0.01). The dose of 150 µg of brFSH (2.58±0.39) also yielded a higher number of blastocysts per OPU (P=0.07) compared to 100 µg of brFSH (1.58±0.30) and pFSH (1.46±0.25). Additionally, the study assessed the pregnancy rate per embryo transfer (P/ET) of *in vitro*-produced embryos derived from Holstein donors who received a single dose of 100 µg of brFSH three days prior to OPU. These embryos were subsequently transferred to lactating Holstein recipients. The results revealed a significant increase in the P/ET among recipients who received embryos from brFSH-treated donors compared to the control group [Control=27.1% (39/144) vs. brFSH=35.1% (60/171); P=0.06]. Based on these findings, the authors concluded that the utilization of a single brFSH treatment for superstimulation not only simplifies donor management but also enhances the efficiency of OPU/IVEP procedures in lactating Holstein cows.

## Recombinant treatment (eCG-Like) for FTAI protocols

Equine chorionic gonadotropin or eCG is an important hormone produced by the placenta of pregnant mares. This hormone is commonly used to enhance the reproduction of beef and dairy heifers and cows, sheep, goats, and pigs. The eCG is a glycoprotein secreted by the endometrial cups of pregnant mares between days 40 and 130 of gestation. This hormone exhibits a half-life of approximately 46 hours. Acting as a gonadotropin, eCG can bind to both FSH and LH receptors and demonstrates FSH and LH-like activities in cattle (Murphy and Martinuk, 1991). Consequently, eCG binds to these follicular receptors and promotes growth and maturation, thereby enhancing the ovulation capacity of the dominant follicle (Stewart and Allen, 1981; Baruselli et al., 2008a). The eCG molecule is composed of two subunits (α - composed of 96 amino acids; and β - composed of 149 amino acids). One of the notable features of eCG is the abundance of carbohydrates, particularly sialic acid (n-acetyl-neuraminic acid), primarily found in the β subunit of the eCG molecule. The abundance of sialic acid contributes to eCG prolonged half-life (Murphy and Martinuk, 1991). Additionally, eCG possesses a high molecular weight and carries a negative charge due to the presence of sialic acid, which hampers its glomerular filtration, further extending its half-life (Legardinier et al., 2005).

Several authors have observed the positive effects of the treatment with eCG on follicular development in cattle during estrous synchronization programs (Bó et al., 2003; Baruselli et al., 2004c; Sá et al., 2010a; Sales et al., 2011). In a previous study conducted by our research group, we observed that the effect of eCG was particularly pronounced in specific groups of animals, including anestrous or undernourished suckled beef cows (Baruselli et al., 2003; Baruselli et al., 2004a; Bó et al., 2007), prepubertal and pubertal beef heifers (Baruselli et al., 2004b; Sá et al., 2010b; Pessoa et al., 2016), primiparous beef cows (Sales et al., 2016), dairy cows in anestrous or with a low body condition score at the beginning of the FTAI protocol (Souza et al., 2009) and in lactating buffaloes submitted to FTAI during the non-breeding season (Carvalho et al., 2013). Treatment with eCG at the time of P4 device removal has been used to enhance fertility in both beef (Baruselli et al., 2004c; Dias et al., 2009; Núñez-Olivera et al., 2014) and dairy cows (Souza et al., 2009; Garcia-Ispierto et al., 2012). This treatment stimulates follicular growth, ovulation, and the function of the corpus luteum (CL), leading to improved reproductive outcomes. Additionally, we observed that eCG treatment promotes conceptus development in cows (Costa e Silva et al., 2013).

Furthermore, eCG induces morpho-functional changes in the CL of treated cattle, including increased CL volume and elevated plasma concentration of progesterone. There were indications of cellular changes related to increased hormonal production and increased CL volume in treated females (Rigoglio et al., 2013). Additionally, it was observed that stimulatory treatment with eCG increased to density and volume of small and large luteal cells and increased the mitochondrial density (Rigoglio et al., 2013). In another study, we analyzed by microarray the effects of stimulatory treatments using eCG on the luteal gene expression profile. It has been observed that eCG causes changes in the expression of multiple genes, particularly those related to P4 synthesis, metabolism, cell differentiation, proliferation, and angiogenesis (Fátima et al., 2013; Moura et al., 2015). Moreover, among the differentially expressed genes after eCG treatment, many were involved in lipid biosynthesis and progesterone production, such as PPARG, STAR, prolactin receptors, and follistatin (Fátima et al., 2013). Also, our results suggest that eCG induces IGF-1 production in the CL, supporting its responsiveness to gonadotropins and the increase in progesterone production by increased lipogenic activity, angiogenesis, and plasticity of the extracellular matrix (ECM;Sousa et al., 2016).

Sales et al. (2011) observed an increase in the diameter of the largest follicle at the time of FTAI (Control=12.9±0.3 mm vs. eCG=13.9±0.2 mm; P=0.006) and on the follicular growth rate between P4 device removal and FTAI (Control=0.95±0.1 mm/day vs. eCG=1.40±0.1 mm/day; P=0.006) in beef cows treated with eCG. In Nelore heifers, Baruselli et al. (2004c) found a positive effect of the treatment of eCG on ovulation rate (Control=50.0% vs. eCG=76.2%; P<0.05), however, the eCG treatment did not interfere with the interval between P4 device removal and ovulation (Control=72.0±2.5 h vs. eCG=72.0±3.1 h). Moreover, treatment with eCG presented an increase in plasma P4 concentrations during diestrus (12 days after the treatment) in both Nelore heifers (Control=2.2±0.2 ng/mL vs eCG=4.3±0.6 ng/mL; Baruselli et al., 2004c) and crossbred beef suckled primiparous (Control=6.4±0.5 ng/mL vs eCG=8.6±0.4 ng/mL; Marques et al., 2003). In another study conducted by Sá et al. (2010b) on cyclic or acyclic Nelore heifers, the administration of eCG at P4 device removal led to an increase in the diameter of the largest follicle at the time of FTAI (48 hours later; Control=9.5±0.2 mm vs. eCG=10.6±0.2 mm; P=0.003), a higher growth rate of the largest follicle from the day of P4 device removal to FTAI (Control=0.64±0.10 mm/day vs. eCG=1.14±0.10 mm/day; P=0.0009) and an enhanced ovulation rate (Control=73.6% vs. eCG=94.4%; P=0.006). Thus, the use of eCG has been found to promote increased follicular growth and ovulation while also raising the progesterone concentration during the diestrus phase following the estrus synchronization. As a result, eCG is currently being successfully employed in FTAI programs.

The use of eCG has also been studied for *in vivo* and *in vitro* superstimulation and embryo production (Bó and Mapletoft, 2014). Our research group studied the hypothesis that satisfactory embryo production could be achieved by administering a single dose of eCG for superovulation in Nelore and Holstein donor cows (Baruselli et al., 2008b). The findings showed that treatment with eCG (1,500 IU or 2,000 IU for Nelore and 2,000 IU or 2,500 IU for Holstein) resulted in a similar number of transferable embryos compared to donors treated with eight gradually decreasing doses of pFSH. These studies provide evidence that incorporating eCG treatment for superstimulation during the synchronization protocol for follicular growth and ovulation can reduce management without compromising embryo production, both in Nelore (*Bos indicus*) and Holstein (*Bos taurus*) donors.

Additionally, we assessed the impact of eCG treatment (administered two days before OPU) on the success of OPU/IVEP programs in Nelore (*Bos indicus*), Brangus (crossbred), and Holstein (*Bos taurus*) donors (Martins et al., 2012). Overall, the eCG treatment resulted in a higher number of viable oocytes in Brangus and Holstein donors. However, it only led to a greater total number of blastocysts per OPU session in Holstein donors, indicating a breed-specific effect on the response to eCG treatment for *in vitro* embryo production.

Currently, the eCG products available in the international market are derived from the blood of pregnant mares. It is essential to note that the glycosylation profile of eCG significantly affects its half-life and effectiveness, and this profile may vary among mares and at different stages of gestation (González-Menció et al., 1978; Manning et al., 1987; Murphy and Martinuk, 1991). However, through genetic engineering techniques, it is possible to produce eCG in laboratory settings without the need for animal-derived products. This advancement has led to the development of recombinant eCG (reCG), which offers a compelling alternative to conventional eCG (Villarraza et al., 2021). By using reCG, not only is the production source better controlled, but also batch-to-batch consistency and reproducibility are ensured (McClamrock, 2003). This represents a significant step forward in the field of reproductive technologies.

Crispo et al. (2021) reported the superovulation response and embryo development in mice obtained with a new glycoprotein with eCG-like activity (reCG) produced by recombinant DNA technology. A total of 150 females from three different mouse strains (C57BL/6J, BALB/Cj, and B6D2F1/J) were subjected to a superstimulatory protocol consisting of 5 IU of natural eCG, 5 IU of reCG or received a placebo (no-eCG), followed by 5 IU of human chorionic gonadotropin 48 hours later. Overall, no significant differences were observed in the total number of zygotes (33.6±2.4 vs. 28.7±2.6) and viable zygotes (31.5±2.4 vs. 25.8±2.5) collected per female between eCG and reCG treated females, respectively, which were greater (P<0.05) than those obtained in no-eCG treated females (6.9±0.7 and 5.9±0.7, respectively). Zygotes derived from the three experimental groups were subjected to *in vitro* culture until hatching 4.5 days post-coitus (DPC). Regardless of the mouse strain, no differences were observed among eCG and reCG-treated females for an overall cleavage rate of 1.5 DPC (58.5% vs. 60.5%), development rate of 3.5 DPC (47.2% vs. 48.9%) and hatching rate of 4.5 DPC (49.5% vs. 54.5%). Control females from no-eCG treated group showed lower cleavage and development rates [36.4% (cleavage rate at 24 hours) and 29.7% (blastocysts at 3.5 DPC; P<0.05). This study demonstrates a comparable superovulation response and embryo development between recombinant and natural eCG treatment.

Villarraza et al. (2021) reported the development of a highly efficient process for the production of reCG in CHO-K1 cells using lentiviral vector systems as a delivery method. The authors found that reCG demonstrated biological activity in cattle since around 30 mg of reCG was needed to exert the same biologic effect as 400 IU of eCG in an ovulation synchronization protocol. Furthermore, the ovulation rate after the synchronization protocol was significantly greater (P<0.05) for both eCG-treated groups (conventional and reCG) than in cows in the control group (no eCG treatment). However, the interval between P4 device removal and ovulation was earlier (P<0.05) in cows treated with reCG than in cows treated with conventional eCG. Finally, the authors found that the CL diameter 13 days after ovulation did not differ among groups.

This information was confirmed by Bó and Cattaneo (2022) in Argentina. The authors used reCG in suckled beef cows submitted to an estradiol (E2) and progesterone (P4)-based FTAI protocol. In experiment 1, Angus and Angus x Hereford beef cows (n=1,244), with 45 to 60 days post-partum, were used to study different doses of reCG [105 IU (1.5 Ml) or 140 IU (2 Ml)] at the time of P4 device removal. In experiment 2, crossbred (*Bos taurus* x *Bos indicus*) beef cows (n=905), with 40 to 90 days post-partum, were used to study different doses of reCG [84 UI (1.2 Ml), 105 UI (1.5 Ml) or 126 UI (1.8 Ml)] at the time of P4 device removal. In experiment 1, P/AI was greater (P<0.05) in cows treated with reCG than in those in the control group [105 UI reCG=52.3% (216/413) vs. 140 IU reCG=53.5% (224/419) vs. Control=44.4% (183/412)]. In experiment 2, although differences among groups only tended to differ [84 IU reCG=38.6% (78/202) vs. 105 IU reCG=38.5% (100/260) vs. 126 IU reCG=36.8% (84/228) vs. Control=27.9% (60/215); P=0.1], there was a significant effect of giving or not reCG, regardless of dose, on P/AI [reCG=38% (262/690) vs. Control=27.9% (60/215); P<0.01]. The authors concluded that treatment with reCG increased P/AI in suckled cows submitted to E2/P4-based FTAI protocols.

In Brazil, our research group also compared the effect of equine chorionic gonadotropin-like glycoprotein produced by recombinant technology (reCG) treatment on the follicular dynamics and pregnancy rate of Nelore cows submitted to FTAI (Abreu et al., 2023). A total of 1,928 suckled *Bos indicus* (Nelore) cows were homogenously distributed either one of the treatments on the day of P4 device removal (D8): 1) cows without eCG treatment; 2) cows treated with 300 IU of conventional eCG and 3) cows treated with 300 IU of reCG. The diameter of the largest follicle was greater (P=0.008) in reCG (11.0±0.2 mm) than in the control group (9.7±0.2 mm). However, no difference was observed between conventional eCG (10.2± 0.2mm) and reCG. Treatment with reCG increased (P<0.0001) the daily growth rate of the largest follicle (1.41±0.10 mm/day) compared to control (0.80±0.09 mm/day) and conventional eCG (1.05±0.08 mm/day) groups. The ovulation rate (presence of CL on D17) was greater (P=0.07) in reCG (51.4%) and in conventional eCG (46.8%) than in the control group (37.0%). The P/AI was greater (P<0.0001) in reCG (39.0%) and in conventional eCG (36.0%) than in the control group (23.0%). In conclusion, reCG has shown to enhance the reproductive performance of cows submitted to FTAI. Interestingly, no significant differences were observed in reproductive efficiency between the conventional eCG and the reCG. This finding highlights the promising potential of this advanced technology in improving reproductive outcomes in cattle breeding programs.

## Recombinant treatment (bST) for artificial insemination and embryo transfer

Bovine somatotropin (bST), also known as bovine growth hormone (bGH), is an endogenous hormone present in cows that plays a vital role in regulating growth and lactation. In the early stages of research, bST was isolated and purified from the bovine pituitary through extraction methods (Evans and Simpson, 1931). The first studies conducted in the 1930s explored the use of crude extracts of bovine somatotropin to evaluate its impact on milk production, leading to promising results that demonstrated improvements in galactopoiesis (Asimov and Krouze, 1937). However, the challenges associated with large-scale extraction hindered the widespread adoption of this therapy. To overcome this limitation, scientists turned to biotechnology to produce synthetic versions of bST, known as recombinant bovine somatotropin (rbST). Recombinant bST, produced through genetic engineering techniques, is a replica of the natural hormone found in cows. The first study using rbST was described in 1982 (Bauman, 1999). Subsequent studies have consistently demonstrated its effectiveness in increasing milk production (West et al., 1990; Zhao et al., 1992; Phipps et al., 1997), while extensive research has confirmed its safety for both human and bovine health (Bauman, 1992; Laurent et al., 1992). In 1993, after rigorous evaluation of its potential impact on human and cattle health, the Food and Drug Administration (FDA) approved the use of rbST (Bauman, 1999; Raux et al., 2022). Currently, more than 20 countries use rbST to increase the efficiency of milk production (Raux et al., 2022), contributing to a more efficient and sustainable livestock industry.

The use of rbST not only exerts a positive impact on milk production but has also garnered significant interest within the scientific community regarding its effects on reproductive efficiency. Similar to bGH, rbST increases the plasmatic levels of IGF-1 and insulin in cattle (Bilby et al., 2004; Cooke et al., 2013; Mercadante et al., 2016), crucial hormones for reproductive processes (Velazquez et al., 2008). The components of the IGF system play vital roles in bovine follicular development and CL function. Several studies support the importance of IGF-1 in the endocrine regulation of ruminant ovaries. When there is a decrease in plasma concentrations of IGF-1 due to a growth hormone receptor deficiency (GHRD), it can lead to a reduction in the number of small antral follicles and the development of the dominant follicle in the first wave, ultimately affecting reproductive efficiency (Chase et al., 1998). Additionally, IGF-1 is involved in the regulation of plasma progesterone concentrations, another critical factor for reproduction (Chase et al., 1998). In the CL, IGF-1 indirectly influences angiogenesis by stimulating VEGF-A production in luteal cells, thereby promoting the proliferation and differentiation of luteal and endothelial cells (Schams et al., 2001). These interactions between IGF-1, hormonal regulation, and angiogenesis in the ovary and CL underscore the significance of this system in ruminant reproductive function. Moreover, it is important to highlight that the effects of rbST treatment on reproduction are dose-dependent and circulating levels of IGF-1 can positively or negatively influence outcomes (Thatcher et al., 2006; Oosthuizen et al., 2018).

Research studies have been conducted to investigate the impact of rbST treatment on reproduction, particularly in high-producing dairy cows undergoing FTAI protocols. Several studies have reported an increase in fertility among cows treated with rbST (Moreira et al., 2000; Moreira et al., 2001; Santos et al., 2004; Ribeiro et al., 2014). Specifically, when lactating cows were administered 500 mg of rbST every 14 days alongside synchronization protocols, an improvement in P/AI was observed (Moreira et al., 2000; Moreira et al., 2001; Santos et al., 2004). Furthermore, the treatment with 500 mg of rbST at the time of estrus detection and 10 days later increased the P/AI in repeat breeder cows (Morales-Roura et al., 2001).

Current research is prioritizing and extensively investigating the use of reduced doses of rbST (below 500 mg) due to concerns that high doses of rbST may elevate circulating concentrations of IGF-1, potentially leading to negative effects on reproduction (Thatcher et al., 2006). These studies aim to determine the optimal dosage of rbST that can enhance reproductive outcomes without compromising fertility in cattle. A study conducted with 1,483 lactating Holstein cows at 50 days postpartum revealed an improvement in fertility (greater P/AI, reduced pregnancy losses, and increased conceptus development) when 325 mg of rbST on the day of artificial insemination and 14 days later were administrated (Ribeiro et al., 2014). Recently, in a study conducted with 834 crossbred beef cows (Rebeis et al., 2023 in press), administration of 325 mg of rbST at the beginning of the synchronization protocol for FTAI increased the pregnancy rate [rbST-D0=53.0% (232/438) vs. Control=48.8% (190/389); P=0.08]. These results were also observed in non-lactating multiparous crossbred cows treated with 500 mg of rbST at the beginning of the synchronization protocol for FTAI (Kaminski et al., 2019). In treated cows with rbST larger diameter of pre-ovulatory follicle (rbST=14.5 mm vs. Control=12 mm; P<0.03), greater ovulation rate (rbST=90.9% vs. Control=69.2%; P=0.09) and greater CL diameter 5 days after FTAI (rbST=19.7 mm vs. Control=16.9 mm; P<0.01) were found by these authors. Starbuck et al. (2006) also showed increase in pregnancy rate in lactating cows treated with 500 mg of rbST on the day of insemination. The authors observed an improvement in P/IA in treated Holstein cows with days in milk (DIM) above 100 (60.4% vs. 40.3%; P<0.05), but no effect was found in dairy heifers or in crossbred lactating beef cows.

Recombinant bovine somatotropin treatment has been utilized in OPU/IVEP programs to enhance follicular population and improve oocyte quality prior to aspiration. Treatment with bST has been shown to increase the follicular population in *Bos taurus* (Gong et al., 1993), *Bos indicus* (Buratini et al., 2000), and buffalo (Sá et al., 2009). Recombinant bST treatment has been found to increase the number of follicles in lactating Holstein cows and the size of the second largest follicle in both lactating and non-lactating cows (De la Sota et al., 1993). Additionally, a study by Lucy et al. (1993) demonstrated that lactating dairy cows treated with bST before day 12 of the estrous cycle, specifically during the first follicular wave (with estrus considered as day 0), exhibited a higher number of ovarian follicles ranging from 3 to 9 mm in size compared to cows treated with saline. Moreover, Buratini et al. (2000) observed a significant increase in plasma IGF-1 concentration and an increase of 36% in the number of small follicles (<5 mm) when *Bos indicus* heifers were treated with rbST on day 3 of the estrous cycle. However, no effect was observed on the number of medium (5-9 mm) or large (>9 mm) follicles. Positive outcomes of rbST treatment include enhancing oocyte quality and embryo development capacity, improving *in vitro* oocyte maturation and increasing fertilization (Pavlok et al., 1996; Bols et al., 1998; Tripp et al., 2000; Roth et al., 2002). We evaluated the effect of 500mg of rbST administered 5 days before OPU on the success of OPU/IVEP programs in Nelore (*Bos indicus*), Brangus (crossbred) and Holstein (*Bos taurus*) donors (Martins et al., 2012). Overall, the bST treatments resulted in a higher number of total blastocysts per OPU session only in Holstein donors. We performed other studies that confirmed the positive effect of rbST treatment before OPU/IVEP increasing embryo production also in prepubertal Holstein heifers. Currently, *in vitro* embryo production with oocytes from heifer calves combined with genomic selection provides a powerful technology platform to reduce generation interval and significantly increase the rate of genetic gain in cattle (Baruselli et al., 2016; Baruselli et al., 2021). rbST treatment increased the cleavage rate, and there was also a tendency for bST to increase the blastocyst rate and the number of blastocysts per OPU in prepubertal heifers (Elliff, 2020). The treatment with bST increased the circulating levels of IGF-1, which may have positively influenced oocyte competence and *in vitro* embryo development (Bevers et al., 1997; Elliff, 2020).

Moreover, investigations have shown positive effects on fertility in studies involving embryo recipients treated with rbST. For instance, Rebeis et al. (2022) observed improved fertility in crossbred heifers recipients (n=751) who received 325 mg of rbST upon removal of the P4 device, with a significant increase in the pregnancy per transfer rate (P/TE) compared to the control group [Control=27.4% (87/317) vs. bST=38.3% (119/311); P=0.004]. In another study by Rebeis et al. (2023 in press), heifers recipients treated with 325 mg of rbST at the beginning of the synchronization protocol (D0) also exhibited enhanced P/TE rates [rbST-D0=57.7% (60/104) vs. Control=48.2% (54/112); P=0.07]. However, it is important to consider that factors such as age, genetics, nutritional status, and overall health, can influence individual cow responses to rbST treatments.

## Conclusions

The utilization of recombinant hormones in cattle reproduction presents a promising alternative to conventional methods of hormone extraction from animal-derived sources. Currently, gonadotropin products used in cattle are derived from pituitary tissue, blood, or urine, requiring extraction and purification processes. However, through genetic engineering techniques, these desired hormones can be synthesized in laboratory settings, eliminating the need for animal-derived materials. This approach offers several advantages, including reduced risk of pathogen transmission and enhanced quality control of hormone products. Additionally, recombinant gonadotropins can be engineered to have prolonged action, enabling single-dose administration. This provides convenience and efficiency in managing cattle reproduction. While recombinant gonadotropins are already available to some extent in human and animal reproduction, further development and licensing of extended-action recombinant hormones are anticipated in the near future. The adoption of recombinant hormones in cattle reproduction offers safer, more reliable, and potentially more convenient options for producers.
